# MCA1 and MCA2 Are Involved in the Response to Hypergravity in Arabidopsis Hypocotyls

**DOI:** 10.3390/plants9050590

**Published:** 2020-05-05

**Authors:** Takayuki Hattori, Yasuhiro Otomi, Yohei Nakajima, Kouichi Soga, Kazuyuki Wakabayashi, Hidetoshi Iida, Takayuki Hoson

**Affiliations:** 1 Department of Biology, Graduate School of Science, Osaka City University, Sumiyoshi-ku, Osaka 558-8585, Japan; d20sc005@dt.osaka-cu.ac.jp (T.H.); o_mix_episode2@msn.com (Y.O.); d4c.ballbreaker.29@gmail.com (Y.N.); soga@sci.osaka-cu.ac.jp (K.S.); wakaba@sci.osaka-cu.ac.jp (K.W.); 2 Department of Biology, Tokyo Gakugei University, Koganei, Tokyo 184-8501, Japan; iida@u-gakugei.ac.jp

**Keywords:** Arabidopsis, gravity resistance, hypergravity, MCA1, MCA2, mechanosensitive channel, signal perception

## Abstract

Plants respond to and resist gravitational acceleration, but the mechanism of signal perception in the response is unknown. We studied the role of MCA (mid1-complementing activity) proteins in gravity perception by analyzing the expression of the *MCA1* and *MCA2* genes, and the growth of hypocotyls of *mca* mutants, under hypergravity conditions in the dark. An *MCA1* promoter::*GUS* fusion reporter gene construct (*MCA1*p::*GUS*) and *MCA2*p::*GUS* were expressed almost universally in etiolated seedlings. Under hypergravity conditions, the expression levels of both genes increased compared with that under the 1 *g* condition, and remained higher, especially in the basal supporting region. On the other hand, *mca*-null and *MCA*-overexpressing seedlings showed normal growth under the 1 *g* condition. Hypergravity suppressed elongation growth of hypocotyls, but this effect was reduced in hypocotyls of *mca*-null mutants compared with the wild type. In contrast, *MCA*-overexpressing seedlings were hypersensitive to increased gravity; suppression of elongation growth was detected at a lower gravity level than that in the wild type. These results suggest that MCAs are involved in the perception of gravity signals in plants, and may be responsible for resistance to hypergravity.

## 1. Introduction

Plants are exposed to a variety of mechanical stresses such as wind and gravity, and they have developed efficient mechanisms to respond to these stressors over evolutionary time, in particular after their emergence on land. We have previously studied the mechanisms of plant responses to gravitational acceleration, mainly with basipetal hypergravity produced by centrifugation [1,2]. Hypergravity generally suppresses elongation growth and increases the rigidity of plant organs. The cell wall is an important source of mechanical strength in plants; it is likely that the properties of the cell wall are also modified under hypergravity conditions. Hypergravity induces changes in cell wall metabolism, such as accumulation of its constituents, polymerization of certain matrix polysaccharides due to breakdown suppression, stimulation of cross-link formation, and modifications to the wall environment, which lead to increased cell wall rigidity [3]. Hypergravity promotes the lateral expansion of plant organs, with concomitant suppression of elongation growth [4]. Cortical microtubules are likely responsible for the anisotropic growth of plant cells [5,6]. Hypergravity induces reorientation of cortical microtubules from transverse to longitudinal directions [4], and increases the expression of tubulin and microtubule-related genes [7,8,9], which may be responsible for the reorientation.

The mechanism of gravity signal perception in the response of plants to hypergravity has been examined. Gadolinium ions, blockers of mechanosensitive ion channels [10,11], nullified the hypergravity-induced modifications to growth anisotropy and cell wall rigidity [12,13]. Hypergravity-induced upregulation of the tubulin- and microtubule-related genes was also suppressed by gadolinium ions [8,9]. Moreover, the gadolinium ions decreased the alignment angle of the epidermal cell files and the cortical microtubules of tubulin mutants at 1 *g*, and hypergravity did not affect these factors when the gadolinium ions were present [14]. These results suggest that mechanosensitive ion channels are involved in the plant’s response to hypergravity, and have contributed to the concept of ‘gravity resistance’ [1,2]. However, the mechanosensitive ion channels have yet to be identified at the molecular level. 

Candidates for the mechanosensitive ion channels have been reported in Arabidopsis; MCA (mid1-complementing activity) proteins are one such candidate. The amino acid sequences of the MCA1 (At4g35920) and MCA2 (At2g17780) proteins share 73% homology and partially complement the yeast *mid1* mutant, which lacks a putative Ca^2+^-permeable mechanosensitive channel [15,16]. Both proteins are localized in the plasma membrane, and have a single transmembrane segment, as well as an EF-hand-like motif and a coiled-coil motif in the N-terminal half and a plac8 motif in the C-terminal half [15,17]. Multiple lines of evidence suggest that MCA1 and MCA2 are involved in mechanosensation. MCA1 is required for sensing substrate (agar medium or soil) hardness [15]. MCA1 and MCA2 are responsible for Ca^2+^ influx in yeast cells and Arabidopsis seedlings [15,16], a hypo-osmotic shock-induced increase in the intracellular Ca^2+^ concentration [15,18], and membrane stretch-induced cation currents in *Xenopus laevis* oocytes [19]. Both proteins are also involved in the cold-induced elevation of Ca^2+^ and cold tolerance [20]. In the present study, we examined the role of MCA1 and MCA2 in gravity perception by analyzing the expression of the *MCA1* and *MCA2* genes, and the growth of etiolated hypocotyls of *mca* mutants, under hypergravity conditions.

## 2. Results

### 2.1. Hypergravity-induced Expression of MCA Genes

**Figure 1:**
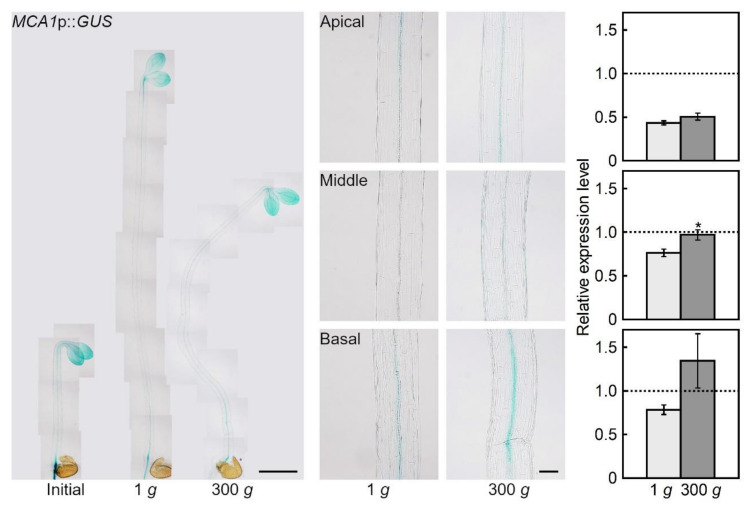
Effects of hypergravity on *MCA1* gene expression. An *MCA1*p::*GUS* transgenic line was grown at 25 °C for 48 h at 1 *g*. The seedlings were then transferred to 1 *g* or 300 *g* conditions and grown at 25 °C for an additional 24 h. Left, GUS-stained seedlings (bar = 1 mm). Middle, magnification of the hypocotyls (bar = 0.1 mm). Right, quantified GUS-staining intensity (expression level) of each region of the hypocotyl. The expression level was normalized to the initial mean value. The dashed line indicates the initial values. Values are means ± SE (*n* = 10–12). * Mean value with a significant difference between the 1 *g* and 300 *g* treatments (Student’s *t-*test: *p* < 0.05).

The expression patterns of *MCA1* and *MCA2* in etiolated seedlings grown at 300 *g* were analyzed using an *MCA1* promoter::*GUS* fusion reporter gene construct (*MCA1*p::*GUS*) and *MCA2*p::*GUS*. *MCA1*p::*GUS* was expressed in the cotyledons and hypocotyls, regardless of age or gravitational conditions (Figure 1). In the hypocotyls, *MCA1*p::*GUS* was strongly expressed in the vascular bundles. Under hypergravity conditions, the expression of *MCA1* increased in the hypocotyls compared to the 1 *g* conditions. *MCA1* expression remained higher in the middle and basal regions.

**Figure 2:**
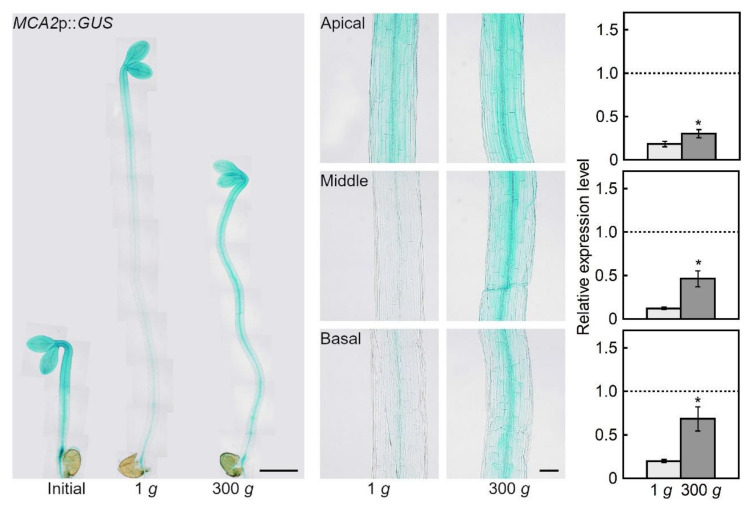
Effects of hypergravity on *MCA2* gene expression. An *MCA2*p::*GUS* transgenic line was grown as described in Figure 1. Left, GUS-stained seedlings (bar = 1 mm). Middle, magnification of the hypocotyls (bar = 0.1 mm). Right, quantified GUS-staining intensity (expression level) of each region of the hypocotyl. The expression level was normalized to the initial mean value. The dashed line indicates the initial values. Values are means ± SE (*n* = 10–12). * Mean value with a significant difference between the 1 *g* and 300 *g* treatments (Student’s *t-*test: *P* < 0.05).

*MCA2*p::*GUS* was also expressed in the cotyledons and hypocotyls (Figure 2). In the hypocotyls, *MCA2*p::*GUS* was expressed in the vascular bundles and the parenchymal and epidermal cells. The expression levels decreased from the apical to the basal regions of the hypocotyls. Under hypergravity conditions, *MCA2* expression increased in all regions of the hypocotyls compared to 1 *g,* and remained higher, particularly in the basal region. 

### 2.2. Hypergravity-Induced Growth of Mca-Null and MCA-Overexpression Mutants

**Figure 3:**
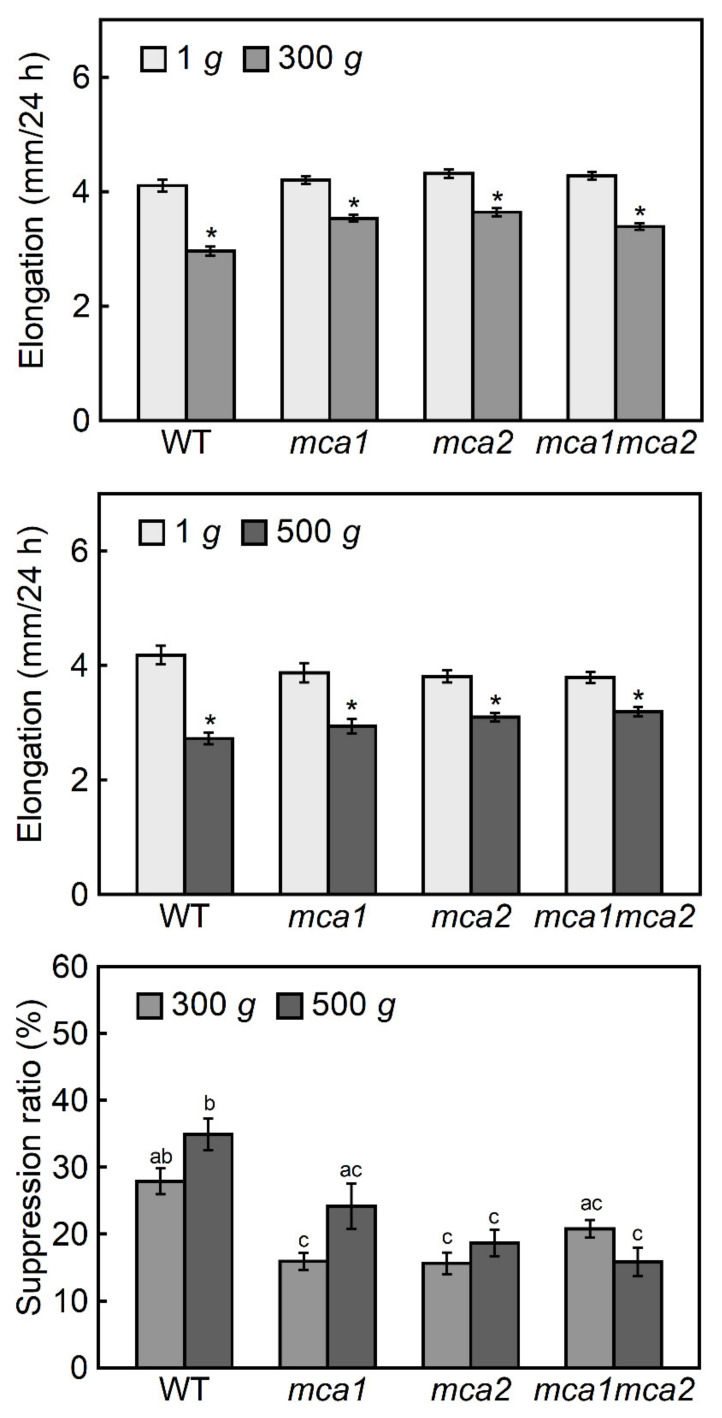
Effects of hypergravity on the elongation growth of hypocotyls in *mca*-null mutants. Wild type (WT) and *mca*-null mutants were grown at 25 °C for 48 h at 1 *g*. The seedlings were then transferred to 1 *g*, 300 *g*, or 500 *g* conditions and grown for an additional 24 h at 25 °C. The length of the hypocotyls was measured using a scale. Values are means ± SE (*n* = 20). Upper, elongation under 1 *g* or 300 *g* conditions after 24 h growth. Middle, elongation under 1 *g* or 500 *g* conditions after 24 h growth. * Mean value with a significant difference between the 1 *g* and hypergravity treatments in each line (Student’s *t-*test: *p* < 0.05). Lower, suppression ratio (the difference between the elongation under 1 *g* and hypergravity conditions was shown as a percentage of the 1 *g* value). Different letters above the bars represent statistically significant differences (Tukey’s HSD test: *p* < 0.05).

The growth of the etiolated hypocotyls of *mca*-null and *MCA*-overexpressing mutants under hypergravity conditions was analyzed to assess if MCA1 and MCA2 are involved in gravity perception, in the response to increased gravity. The growth of the *mca1*-null, *mca2*-null, and *mca1*-null *mca2*-null double mutants (double mutant) was normal in the dark at 1 *g* (Figure 3), although the double mutant showed growth defects in the light [16]. Under hypergravity conditions at 300 *g*, elongation growth of the hypocotyls was suppressed by 30% in the wild type. Hypergravity at 300 *g* also suppressed elongation growth of the hypocotyls in the *mca1*-null, *mca2*-null, and double mutants. However, the suppression ratio of the three *mca*-null mutants was smaller than that of the wild type, and was 16%–21% (Figure 3). There were no differences in the growth suppression ratios of the three *mca*-null mutants. In a higher gravity condition at 500 *g*, growth suppression was slightly enhanced in the wild type and *mca1*-null mutant, but not in the *mca2*-null or double mutants (Figure 3). The level of elongation growth suppression by 500 *g* hypergravity was similar between the *mca1*-null, *mca2*-null, and double mutants.

**Figure 4:**
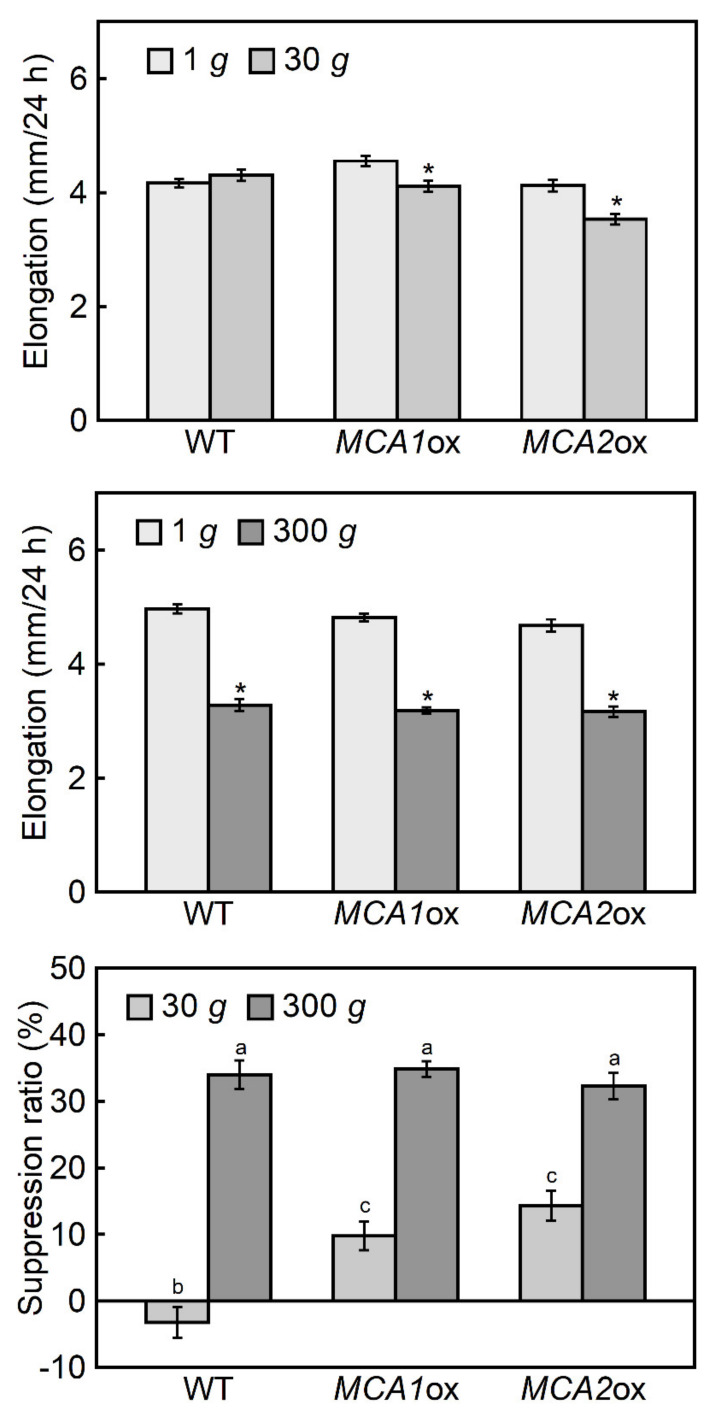
Effects of hypergravity on the elongation growth of hypocotyls in *MCA*-overexpressing mutants. Wild type (WT) and *MCA*-overexpressing mutants (*MCA1ox* and *MCA2ox*) were grown on agar medium at 25 °C for 48 h at 1 *g*. Seedlings were then transferred to 1 *g*, 30 *g,* or 300 *g* conditions and grown for an additional 24 h at 25°C. The length of the hypocotyls was measured using a scale. Values are means ± SE (*n* = 20). Upper, elongation under 1 *g* or 30 *g* conditions after 24 h growth. Middle, elongation under 1 *g* or 300 *g* conditions after 24 h growth. * Mean value with a significant difference between the 1 *g* and hypergravity treatments in each line (Student’s *t-*test: *p* < 0.05). Lower, suppression ratio (the difference between the elongation under 1 *g* and hypergravity conditions was shown as a percentage of the 1 *g* value). Different letters above the bars represent statistically significant differences (Tukey’s HSD test: *p* < 0.05).

Seedling growth of the *MCA1*-overexpressing and *MCA2*-overexpressing mutants in the dark at 1 *g* was normal (Figure 4), but the *MCA1*-overexpressing mutant showed growth defects in light [15]. At 300 *g*, the elongation growth of the hypocotyls was suppressed by 30%–35%, and there were no differences in the growth suppression ratios of the wild type or *MCA1*- and *MCA2*-overexpressing mutants. Under lower gravity conditions at 30 *g*, the wild type exhibited no elongation growth suppression, but a small significant suppression was observed in the *MCA1*- and the *MCA2*-overexpressing mutants (Figure 4).

## 3. Discussion

In the present study, we used etiolated seedlings to avoid the influence of light—the light response pathway overlaps with the gravity response and acts as a gravity-substitution factor in plant growth regulation [1,21]. *MCA1*p::*GUS* and *MCA2*p::*GUS* were expressed almost universally in the etiolated seedlings (Figure 1 and Figure 2), which is consistent with observations from light-grown seedlings [16]. Under hypergravity conditions, the expression levels of both genes increased compared to 1 *g*. Preliminary microarray analyses showed that the expression levels of *MCA1* and *MCA2* were higher under hypergravity conditions, but lower under microgravity conditions in space compared to 1 *g*. These results indicate that the expression of both genes is regulated by gravitational conditions. The amount of signal receptors affects the sensitivity to a given signal, so plants would likely prepare for gravitational stress by increasing the expression of *MCA1* and *MCA2* to enhance gravity sensitivity.

The level of GUS expression in the *MCA1*p::*GUS* line (Figure 1) was lower than in the *MCA2*p::*GUS* line (Figure 2). This result is consistent with the same lines grown under light conditions [16]. However, our observations may not necessarily reflect the actual expression levels of *MCA1* and *MCA2,* because GUS expression often varies from line to line. According to preliminary microarray analyses, the expression level of *MCA1* was several times higher than that of *MCA2* under 1 *g* conditions.

The growth of the etiolated hypocotyls of *mca* mutants was analyzed under hypergravity conditions to investigate the role of MCA1 and MCA2 in gravity perception, in the response to increased gravity. In general, the *mca*-null and *MCA*-overexpressing seedlings grew normally in the dark at 1 *g* (Figure 3 and Figure 4), although some growth defects were observed in light-grown mature plants [15,16]. Under hypergravity conditions, elongation growth was suppressed, but this effect was reduced in the *mca1*-null, *mca2*-null, and double mutants compared to the wild type (Figure 3). Elongation growth suppression was detected at a lower gravity level (30 *g*) in the *MCA1*- and *MCA2*-overexpressing mutants (Figure 4), indicating that the *MCA*-overexpressing seedlings were more sensitive to hypergravity. These results support the hypothesis that MCAs are involved in the perception of gravity when Arabidopsis hypocotyls are subjected to hypergravity.

The suppression of hypocotyl elongation under 300 *g* and 500 *g* hypergravity conditions was similar between the *mca1*-null, *mca2*-null, and double mutants, although the suppression ratio was smaller than that of wild type; all were 45%–70% of the wild type (Figure 3). These results suggest that the functions of MCA1 and MCA2 overlap, and that other mechanosensitive channels are also involved in gravity signal perception. Possible candidates are MSL9 and MSL10, the Arabidopsis homologs of the bacterial mechanosensitive channel of small conductance (MscS), which has mechanosensitive channel activity in the plasma membrane of root cells [22]. The involvement of MSLs in the plant response to hypergravity is an interesting topic for future studies.

Elongation in Arabidopsis hypocotyls occurs in the upper 12 cells of the epidermal cell file, which is suppressed by hypergravity [14]. The results of growth of *mca* mutants (Figure 3 and Figure 4) suggest that MCAs are involved in the perception of gravity in this elongation region. On the other hand, the expression levels of MCAs increased in all regions of the hypocotyls under hypergravity conditions, particularly in the basal region (Figure 1 and Figure 2), which plays a role in supporting the whole plant body. Thus, MCAs may be involved in the gravity perception in all regions of the hypocotyls, and their roles in the supporting region might be especially important for plants.

Here, we analyzed changes in the expression of *MCA* genes and the growth of *mca* mutants in hypergravity conditions. In general, shoot organs grow at half the rate of the 1 *g* control, even at 300 *g* [1]. The growth and cell wall parameters vary in proportion to the logarithm of the magnitude of gravity, and hypergravity at 300 *g* causes about half the changes observed in microgravity in space (ca 10^−4^ *g*) [1]. Moreover, the effects of 300 *g* hypergravity on growth and the cell wall were restored when the plant materials were transferred to 1 *g* conditions, and were completely nullified by the presence of gadolinium ions, as mentioned above. These results indicate that hypergravity at 300 *g* is not an extraordinary stimulus for plants, and that the plant response to this magnitude of gravity stress is within the normal physiological limits. However, the role of MCAs in the perception of gravity signals at 1 *g* has yet to be determined. For this purpose, analyses under true microgravity conditions in space may be most effective as the control; a space experiment termed plant gravity sensing (PI: H. Tatsumi) is now underway.

## 4. Materials and Methods 

### 4.1. Plant Materials and Growth Analysis

The Columbia-0 ecotype of Arabidopsis and its transgenic lines *mca1*-null, *mca2*-null, *mca1*-null *mca2*-null, *MCA1*-overexpressing, *MCA2*-overexpressing, *MCA1*p::*GUS,* and *MCA2*p::*GUS* were used in this study. The details of these lines have been previously described [15,16]. Seeds were sown on 1% (*w/v*) agar in a 50 ml centrifuge tube, incubated at 4 °C in the dark for 4 days, and then exposed to light (50 μmol m^-2^ s^-1^, red: blue = 2:1) at 23 °C for 1 day to induce germination. After germination, the seeds were incubated at 25 °C in the dark for 1 day. Hypergravity experiments were conducted as reported previously [23]. The seedlings were exposed to hypergravity at 30–500 *g* (via centrifugation; H-28-F, Kokusan, Tokyo, Japan) at 25 °C for 1 day in the dark. After treatment, the lengths of the hypocotyls were measured using a scale. The suppression ratio was calculated as a ratio (percentage) of the difference between the elongation under 1 *g* and the hypergravity conditions to the 1 *g* value. Because the lengths of hypocotyls at 1 *g* were different from line to line, the elongation growth data were compared between the 1 *g* and hypergravity treatments in each line and analyzed with Student’s *t-*test (upper and middle panels of Figure 3 and Figure 4). The suppression ratio data were analyzed using the Tukey HSD test (lower panels of Figure 3 and Figure 4).

### 4.2. Analysis of MCA Gene Expression

Seedlings were fixed in 90% acetone in the dark for 1 h and washed twice with 0.1 M sodium phosphate buffer, pH 7.0. The seedlings were then incubated at 37 °C for 20 h with X-Gluc buffer (0.5. mg mL^−1^ 5-bromo-4-chloro-3-indolyl-β-D-glucuronic acid (BMS), 0.1 M sodium phosphate buffer, pH 7.0, 0.5 mM potassium ferrocyanide, and 0.5 mM potassium ferricyanide) after infiltration with a vacuum pump for 1 h. The stained seedlings were fixed in a mixed solution (ethanol: acetic acid = 3:1) for 5 h, washed stepwise with 70%, 50%, and 20% ethanol for 10 min each, and cleared overnight at 4 °C with a cleaning solution (chloral hydrate: glycerol: water = 8:1:3). Quantification of the GUS-staining intensity of each region of the hypocotyl was performed using ImageJ, as reported previously [24]. The GUS-staining intensity data (Figure 1 and Figure 2) were analyzed using Student’s *t*-test.

## 5. Conclusions

MCA1 and MCA2 are at least partly involved in gravity perception when Arabidopsis hypocotyls are exposed to hypergravity. These genes may be responsible for gravity signal perception in the gravity resistance of plants. Future studies should examine the involvement of MCA proteins in plants’ resistance to 1 *g* gravity by space experiments.
